# Peptide-guided JC polyomavirus-like particles specifically target bladder cancer cells for gene therapy

**DOI:** 10.1038/s41598-021-91328-7

**Published:** 2021-06-04

**Authors:** Wei-Hong Lai, Chiung-Yao Fang, Ming-Chieh Chou, Mien-Chun Lin, Cheng-Huang Shen, Chun-Nun Chao, Yeong‐Chin Jou, Deching Chang, Meilin Wang

**Affiliations:** 1 Department of Urology, Ditmanson Medical Foundation, Chiayi Christian Hospital, Chiayi, Taiwan; 2 Department of Medical Research, Ditmanson Medical Foundation, Chiayi Christian Hospital, Chiayi, Taiwan; 3 Institute of Molecular Biology, National Chung Cheng University, 168, University Rd., Min-Hsiung, Chiayi, 621 Taiwan; 4 Department of Pediatrics, Ditmanson Medical Foundation, Chiayi Christian Hospital, Chiayi, Taiwan; 5 Department of Microbiology and Immunology, School of Medicine, Chung-Shan Medical University and Clinical Laboratory, Chung-Shan Medical University Hospital, No. 110, Sec. 1, Jianguo N. Rd., Taichung City, 40201 Taiwan

**Keywords:** Cancer therapy, Bladder

## Abstract

The ultimate goal of gene delivery vectors is to establish specific and effective treatments for human diseases. We previously demonstrated that human JC polyomavirus (JCPyV) virus-like particles (VLPs) can package and deliver exogenous DNA into susceptible cells for gene expression. For tissue-specific targeting in this study, JCPyV VLPs were conjugated with a specific peptide for bladder cancer (SPB) that specifically binds to bladder cancer cells. The suicide gene thymidine kinase was packaged and delivered by SPB-conjugated VLPs (VLP-SPBs). Expression of the suicide gene was detected only in human bladder cancer cells and not in lung cancer or neuroblastoma cells susceptible to JCPyV VLP infection in vitro and in vivo, demonstrating the target specificity of VLP-SPBs. The gene transduction efficiency of VLP-SPBs was approximately 100 times greater than that of VLPs without the conjugated peptide. JCPyV VLPs can be specifically guided to target particular cell types when tagged with a ligand molecule that binds to a cell surface marker, thereby improving gene therapy.

## Introduction

Gene therapy involves the delivery of therapeutic genes into the patient's body to treat disease as an alternative to traditional treatments^1–3^. Since the first gene therapy trial was approved in 1989^4^, nearly 2600 gene therapy trials have been completed or are underway worldwide^5^. The majority of these clinical trials involve treatments for cancer (65.0%), along with treatments for inherited monogenic diseases (11.1%) and infectious diseases (7.0%). However, safely protecting the entry of genes into specific target cells without harming nontarget cells remains the greatest challenge for gene therapy. During the process of evolution, viruses developed a mechanism that specifically protects the entry of viral genes into susceptible cells. Viral vectors derived from viruses retain this property, thus providing an attractive option as gene therapy vectors^6–8^.

We previously reported the first cloning and expression of the major capsid protein VP1 of JC polyomavirus (JCPyV) in insect cells^9^, *Escherichia coli*^10^, and yeast^11^. The recombinant VP1 protein is capable of self-assembly into a virus-like particle (VLP) for gene delivery. As a gene delivery vector, recombinant JCPyV VLPs can be easily generated in large quantities and at low cost. Exogenous genes of interest can be packaged by the VLPs without requiring viral genetic material, which can be subsequently delivered into tissues susceptible to JCPyV to enable gene transduction and gene therapy. These VLPs were successfully used to package and deliver exogenous DNA into human kidney cells for expression^9^. We also used these VLPs to deliver an antisense oligodeoxynucleotide into human glioma (SVG) cells, which resulted in growth inhibition in these cells^12^. More recently, we demonstrated the ability of these VLPs to deliver a suicide gene, thymidine kinase (tk), into human colon carcinoma cells^13^, diffuse large B-cell lymphoma cells^14^, and glioblastoma cells^15^. For tissue-specific gene expression, tissue-specific promoters were constructed to drive the expression of the suicide gene, which was delivered by the VLPs to inhibit the growth of bladder cancer cells^16^, lung adenocarcinoma cells^17,18^, and prostate cancer cells^19^ in a mouse model. Furthermore, the VLPs were used to successfully deliver a BK polyomavirus (BKPyV) LT peptide-specific small hairpin RNA to inhibit BKPyV replication in human kidney cells^20^ and to deliver an interleukin (IL)-10 RNA interference vector into macrophage cells to reduce IL-10 expression^21^ as a possible gene therapy for systemic lupus erythematosus. Moreover, we found that the VLPs could package exogenous DNA molecules of up to 9.4 kb in length^22^. The development of a gene delivery vector using JCPyV VLPs has been reviewed previously^23^. In this study, we extend this development in a further attempt to conjugate these VLPs with a bladder cancer-binding peptide to specifically target bladder cancer cells.

The BC, DE, and HI loops are the exposed domains in the JCPyV VP1 molecule that interact with cell receptors. Therefore, polymorphisms in these regions affect the binding of the virus to host cell receptors and may be associated with viral toxicity or progressive multifocal leukoencephalopathy (PML) development and severity^24–26^. Accordingly, altering or modifying the exposed domains of JCPyV VLPs can change their initial binding ability or affinity for host cell receptors. In recent years, phage display technology has been used to find cancer-specific peptides, which have successfully been used to deliver therapeutic genes or drugs to tumors, thereby specifically killing tumor cells^27^. A specific peptide for bladder cancer (abbreviated SPB; peptide sequence: CSNRDARRC) was discovered by Lee et al*.*^28^ and can be used for the diagnosis or treatment of bladder cancer. In this study, this SPB was conjugated with JCPyV VLPs for the investigation of bladder cancer-specific targeting.

Urothelial carcinoma bladder cancer is the second most common cancer of the urinary tract. Approximately 75% of bladder cancers are non-muscle-invasive bladder cancers (NMIBCs), which are characterized by a high recurrence rate. Approximately 25% of patients have muscle-invasive bladder cancer (MIBC) and must undergo radical cystectomy^29,30^. Thus, patients with NMIBC require regular lifelong invasive cystoscopies to monitor tumor recurrence or progression. In the present study, we used sulfosuccinimidyl 4-(N-maleimidomethyl) cyclohexane-1-carboxylate (sulfo-SMCC) to chemically link JCPyV VLPs and a SPB to form JCPyV VLP-SPBs. We further tested whether JCPyV VLP-SPBs packaging a suicide tk gene can specifically target human bladder cancer cells and inhibit the growth of these cells in vitro and in a mouse xenograft model in vivo.

## Results

### SPB binding specificity

**Figure 1:**
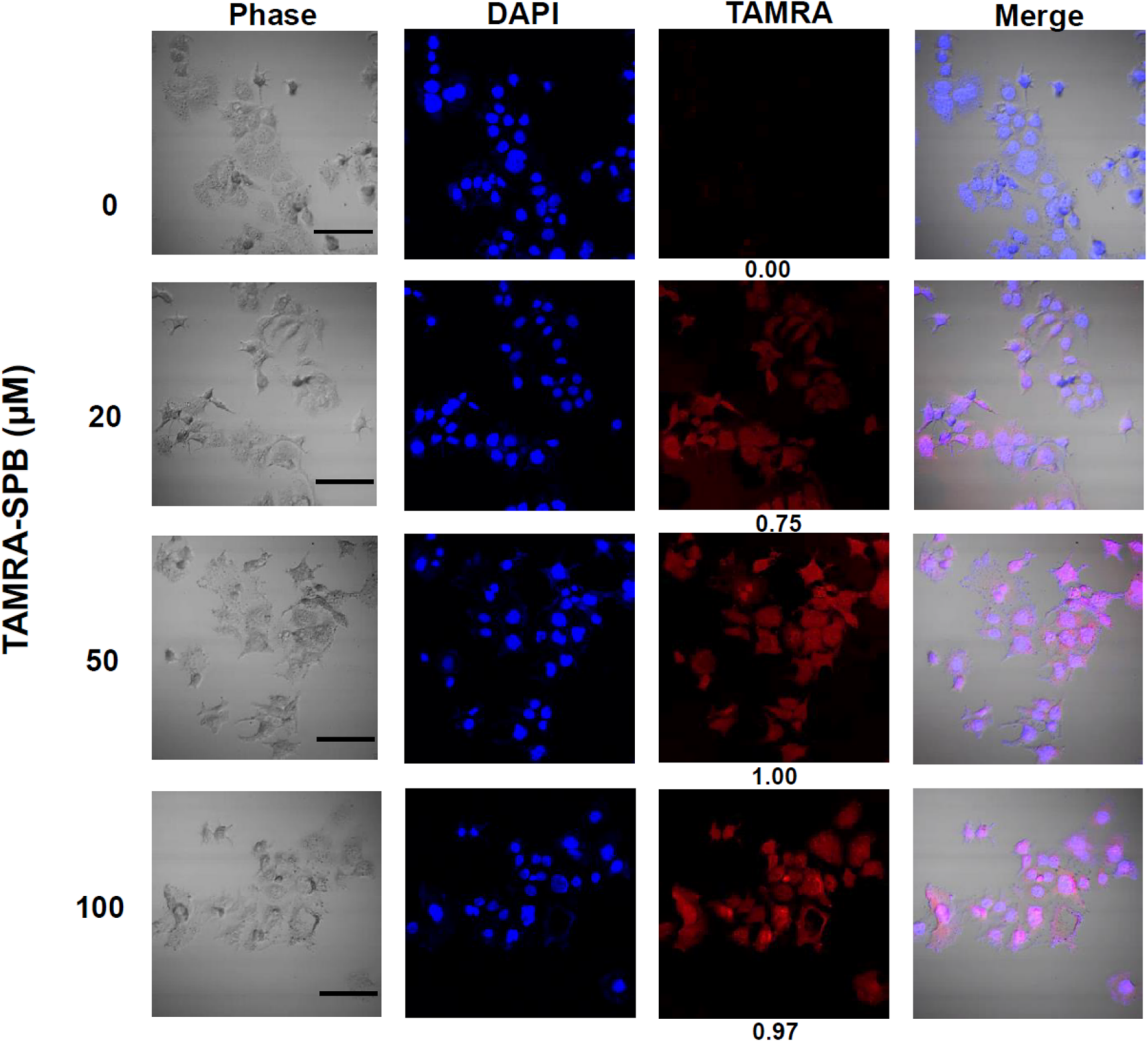
Binding of the bladder cancer-specific peptide (SPB) to HT-1376 bladder cancer cells. The SPB was conjugated with the fluorescent dye TAMRA to form a TAMRA-SPB conjugate, and the binding of TAMRA-SPB to bladder cancer cells was determined by detection of red fluorescence using a confocal microscope. Scale bar: 100 µm.

**Figure 2:**
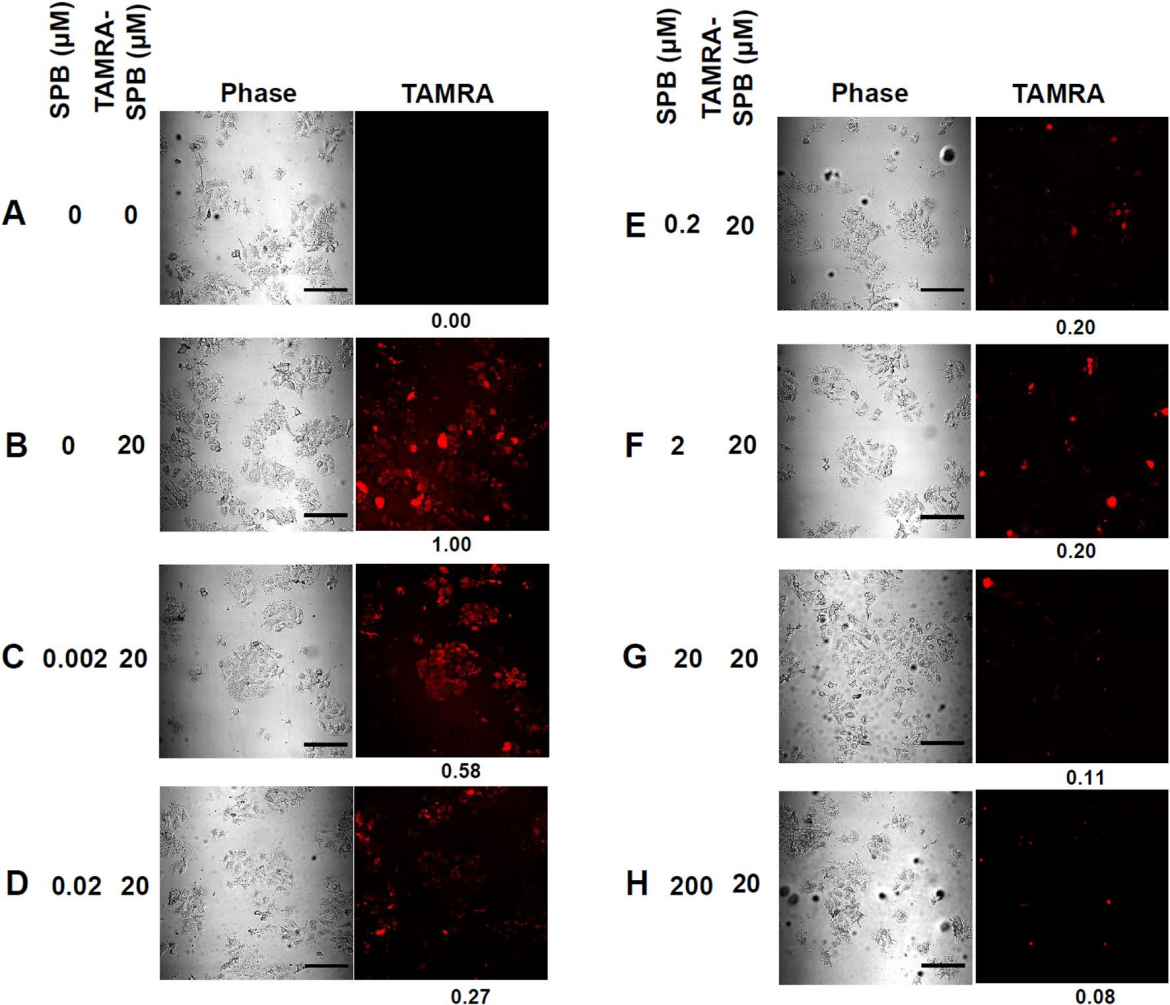
Binding specificity of the SPB to HT-1376 bladder cancer cells. The TAMRA-SPB conjugate was competed with the SPB at different concentrations, and binding was analyzed by detection of red fluorescence using a confocal microscope. The TAMRA-SPB conjugate was competed with the SPB at different concentrations, and binding was analyzed by detection of red fluorescence using a confocal microscope. (**A**) HT-1376 cells not treated with TAMRA-SPB or SPB were used as negative controls. (**B**) HT-1376 cells treated with 20 µM TAMRA-SPB were used as the positive control. HT-1376 cells were treated with 20 µM TAMRA-SPB, and the binding specificity was assessed by competing TAMRA-SPB with (**C**) 0.002 µM SPB, (**D**) 0.02 µM SPB, (**E**) 0.2 µM SPB, (**F**) 2 µM SPB, (**G**) 20 µM SPB, and (**H**) 200 µM SPB. The red fluorescence of TAMRA-SPB was visualized under a confocal microscope. Scale bar: 100 µm.

Previously, a human bladder cancer cell-specific binding peptide (i.e., SPB) was discovered by Lee et al*.*^28^. Here, we first confirmed the binding specificity of this SPB to bladder cancer cells. The SPB was linked to the fluorescent dye tetramethylrhodamine (TAMRA) to form a TAMRA-SPB conjugate. Fluorescence microscopy showed that the red fluorescent SPB (TAMRA-SPB) bound specifically to HT-1376 bladder cancer cells (Fig. 1). As the TAMRA-SPB concentration increased, the red fluorescence of the HT-1376 cells exhibited a concentration-dependent effect (Fig. 1). To further confirm the specificity of TAMRA-SPB binding to HT-1376 cells, we used unconjugated SPB to compete with TAMRA-SPB binding. With increasing concentrations of unconjugated SPB, the red fluorescence of TAMRA-SPB decreased (Fig. 2). This result demonstrated that this SPB can be used to achieve specific binding to bladder cancer cells.

### Binding specificity of JCPyV VLP-SPBs to bladder cancer cells

**Figure 3:**
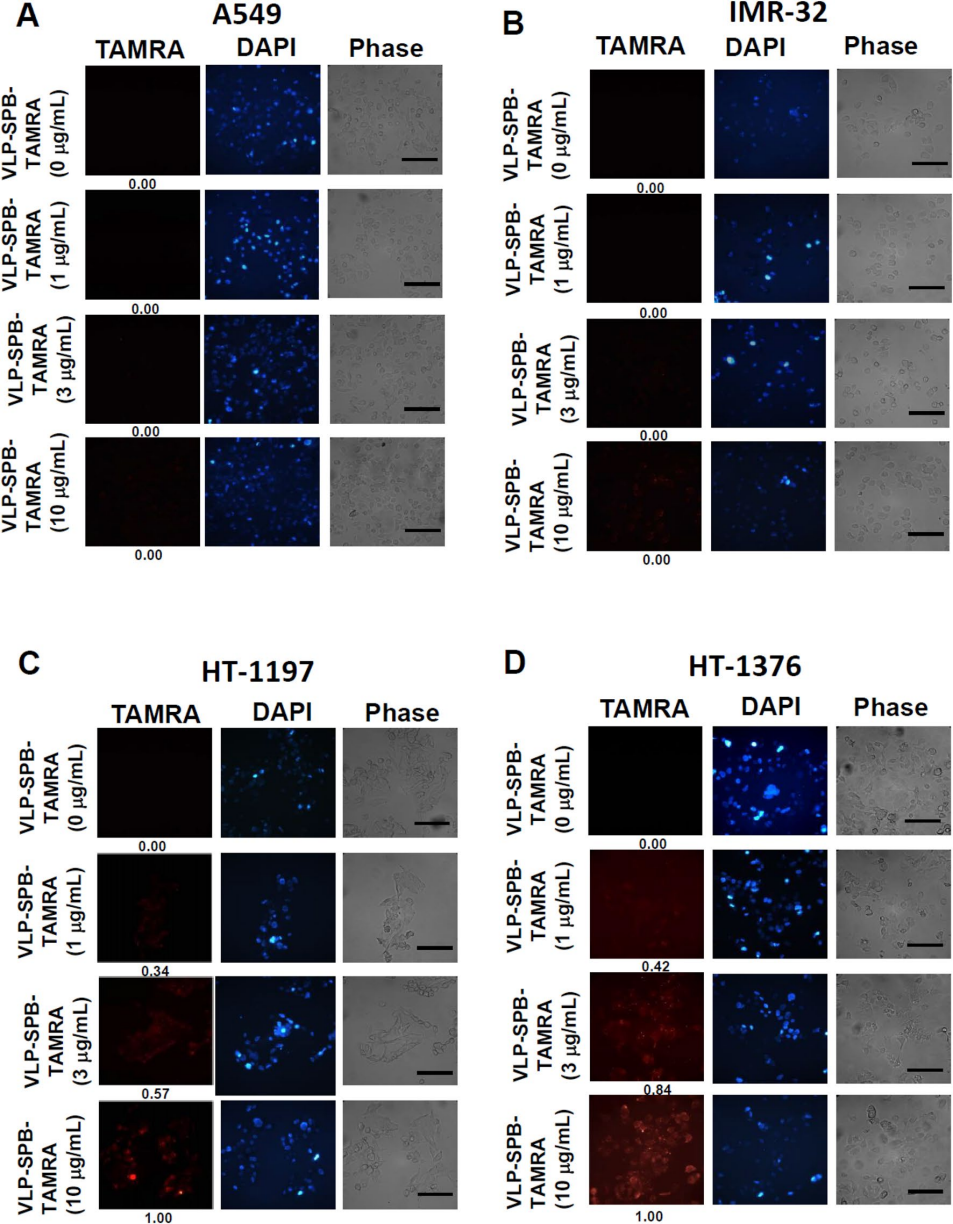
Determination of the binding specificity of JCPyV VLP-SPBs. JCPyV VLP-SPB-TAMRA at different concentrations was used to bind (**A**) A549 lung cancer cells, (**B**) IMR-32 neuroblastoma cells, (**C**) HT-1197 bladder cancer cells and (**D**) HT-1376 bladder cancer cells. The cells were observed under a fluorescent microscope. Scale bar: 100 µm.

To increase the specificity of gene targeting, we attempted to link the SPB peptide and JCPyV VLPs by (sulfosuccinimidyl 4-(N-maleimidomethyl)cyclohexane-1-carboxylate) (sulfo-SMCC) crosslinker. Compared to the X-ray diffraction pattern simulating SV40 VP1 (PDB: 1SVA)^31^, nine amino acids with primary amines are located in outer loops of JCPyV VP1 molecule, allowing stable covalent conjugation with SPB peptide by sulfo-SMCC. We next tested the cell binding specificity of JCPyV VLP-SPBs to bladder cancer cells in comparison to human lung cancer cells (A549) and neuroblastoma cells (IMR-32). JCPyV VLPs conjugated with TAMRA-SPB, namely, JCPyV VLP-SPB-TAMRA, were used to determine the binding specificity of JCPyV VLP-SPBs to bladder cancer cells. When 1 µg, 3 µg, or 10 µg of JCPyV VLP-SPB-TAMRA was separately added to A549 and IMR-32 cells, no red fluorescence representing JCPyV VLP-SPB-TAMRA was observed (Fig. 3A,B). In contrast, slight red fluorescence representing JCPyV VLP-SPB-TAMRA was detected after addition to HT-1197 (Fig. 3C) and HT-1376 (Fig. 3D) bladder cancer cells at low concentrations (1 µg, 3 µg), and obvious red fluorescence was observed in both cell lines when the concentration of JCPyV VLP-SPB-TAMRA was increased to 10 µg (Fig. 3C,D). In our previous study, we have demonstrated that JCPyV VLP can successfully deliver exogenous DNA into lung cancer cells (A549)^17,18^**,** neuroblastoma cells (IMR-32)^12^, and bladder cancer cells (HT-1197)^16^ for gene expressions. After conjugated with bladder cancer specific peptide SPB, JCPyV VLP specifically binds to bladder cancer cells, simultaneously losing its binding to susceptible lung and neuroblastoma cells. This result demonstrates that conjugation with a specific peptide allows alteration of the original tropism of JCPyV VLPs.

### Specific cytotoxicity of JCPyV VLP-SPBs to bladder cancer cells

**Figure 4:**
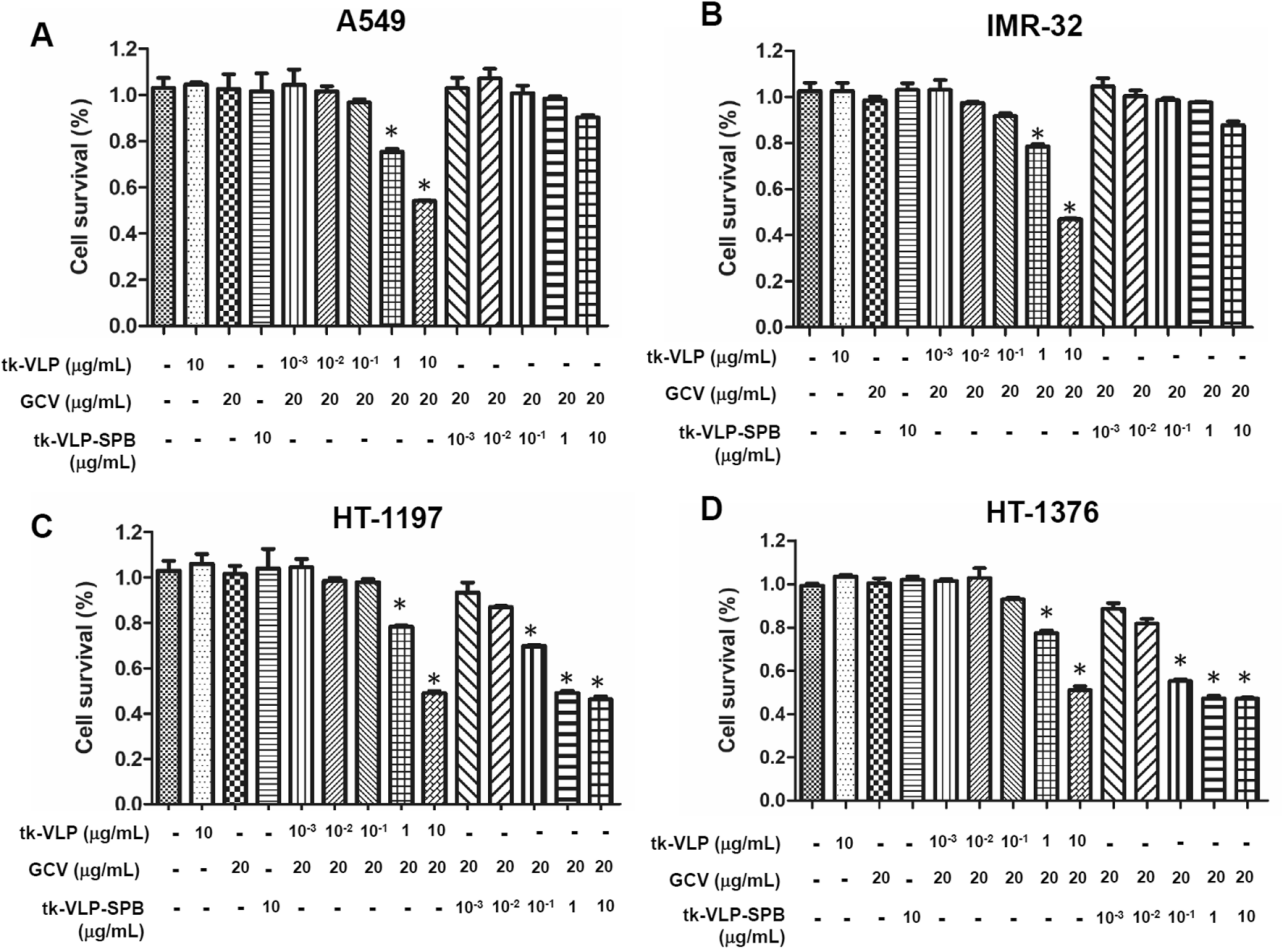
Determination of the specific cytotoxicity of tk-JCPyV VLP-SPBs. Specific cytotoxicity of tk-JCPyV VLP-SPBs to (**A**) A549 lung cancer cells, (**B**) IMR-32 neuroblastoma cells, (**C**) HT-1197 bladder cancer cells and (**D**) HT-1376 bladder cancer cells treated with different concentrations (0.001, 0.01, 0.1, 1, and 10 µg/mL) of tk-JCPyV VLPs or tk-JCPyV VLP-SPBs in the presence of 20 µg/mL ganciclovir (GCV). Cell viability was analyzed using CCK8 reagent. Experiments were triplicated. Kruskal–Wallis test was used to determine the statistics difference. . *: *P* < 0.05 was considered statistically significant and is indicated by asterisks (*).

After confirming that JCPyV VLP-SPBs can bind specifically to bladder cancer cells, we further tested whether JCPyV VLPs conjugated with SPB could selectively deliver the suicide gene tk to inhibit bladder cancer cell growth in the presence of ganciclovir (GCV). When A549 lung cancer cells were treated with different concentrations (0.001, 0.01, 0.1, 1, and 10 µg/mL) of tk-JCPyV VLPs, 1 µg/mL tk-JCPyV VLPs in the presence of the GCV reduced cell viability by approximately 30%, and 10 µg/mL tk-JCPyV VLPs reduced cell viability by approximately 50% (Fig. 4A). However, the survival of A549 cells was nearly unaffected when these cells were treated with different concentrations (0.001, 0.01, 0.1, 1, and 10 µg/mL) of tk-JCPyV VLP-SPBs (Fig. 4A). Similar results were found for IMR-32 neuroblastoma cells. When IMR-32 cells were treated with different concentrations (0.001, 0.01, 0.1, 1, and 10 µg/mL) of tk-JCPyV VLPs, cell viability decreased in a concentration-dependent manner, with approximately 91.7%, 78.5%, and 47.9% viability after treatment with 0.1 µg/mL, 1 µg/mL, and 10 µg/mL tk-JCPyV VLPs, respectively, in the presence of GCV (Fig. 4B). However, treatment with different concentrations (0.001, 0.01, 0.1, 1, and 10 µg/mL) of tk-JCPyV VLP-SPBs did not significantly decrease the viability of IMR-32 cells (Fig. 4B). This result indicates after conjugation with bladder cancer specific peptide SPB, JCPyV VLP failed to transduce tk suicide gene into susceptible lung cancer cells and neutoblasoma cells. The tropism of JCPyV VLP is changed when linked with bladder cancer specific peptide.

Next, we evaluated the cytotoxic effects of tk-JCPyV VLP-SPBs on HT-1197 and HT-1376 bladder cancer cells. When HT-1197 cells were treated with different concentrations (0.001, 0.01, 0.1, 1, and 10 µg/mL) of tk-JCPyV VLPs in the presence of the prodrug GCV, cell viability decreased in a concentration-dependent manner, with 78.3% and 49.1% viability after treatment with 1 µg/mL and 10 µg/mL tk-JCPyV VLPs, respectively (Fig. 4C). Furthermore, even a low concentration (0.01 µg/mL) of tk-JCPyV VLP-SPBs reduced the viability of HT-1197 cells to 86.9%, and the cytotoxic effect gradually increased in a concentration-dependent manner, with 69.8%, 49.2%, and 46.3% viability after treatment with 0.1, 1, and 10 µg/mL tk-JCPyV VLP-SPB, respectively (Fig. 4C). Similarly, when HT-1376 cells were treated with different concentrations (0.001, 0.01, 0.1, 1, and 10 µg/mL) of tk-JCPyV VLPs in the presence of the prodrug GCV, cell viability decreased with 77.3% and 51.1% viability after treatment with 1 µg/mL and 10 µg/mL tk-JCPyV VLPs, respectively (Fig. 4D). Furthermore, the lowest concentration (0.001 µg/mL) of tk-JCPyV VLP-SPBs reduced the viability of another bladder cancer cell line, HT-1376, to 88.7% when administered in combination with GCV. As the concentration of tk-JCPyV VLP-SPBs was gradually increased to 0.01, 0.1, 1, and 10 µg/mL, the viability of HT-1376 bladder cancer cells also gradually decreased to 81.8%, 55.3%, 47.2%, and 47.1%, respectively (Fig. 4D). After linked with bladder cancer specific peptide SPB, in the presence of GCV, 1 µg/mL of tk-JCPyV VLP-SPB treatment exhibits similar growth inhibition effect with10 µg/mL of tk-JCPyV VLP in HT-1197 cells (49.2% compared to 49.1% cell survival, respectively) (Fig. 4C); 0.1 µg/mL of tk-JCPyV VLP-SPB treatment exhibits similar growth inhibition effect with 10 µg/mL of tk-JCPyV VLP in HT-1376 cells (55.3% compared to 51.1% cell survival, respectively) (Fig. 4D). In summary, tk-JCPyV VLP-SPBs had cytotoxic effects specific to bladder cancer cells. These results demonstrated that tk-JCPyV VLP-SPBs greatly enhanced the cytotoxic effects of tk-JCPyV VLPs on bladder cancer cells. Therefore, linking to a specific peptide can increase the tissue specificity and efficiency of JCPyV VLPs to further enhance their ability to deliver genes for expression in specific cells.

### Specific inhibition of bladder cancer cell growth by tk-JCPyV VLP-SPBs in a xenograft mouse model

After testing the specific cytotoxicity of tk-JCPyV VLP-SPBs in bladder cancer cells, we further tested the specificity of tk-JCPyV VLP-SPB cytotoxicity to bladder cancer cells in an animal model. As shown in Fig. 4, linking a specific peptide of bladder cancer cells has changed the tropism of JCPyV VLP to lung cancer cells. We proposed that after conjugated with specific peptide of bladder cancer cells, tk-JCPyV VLP-SPBs will specifically deliver the packaged tk gene into bladder tumor nodules instead of lung tumor nodules.

**Figure 5:**
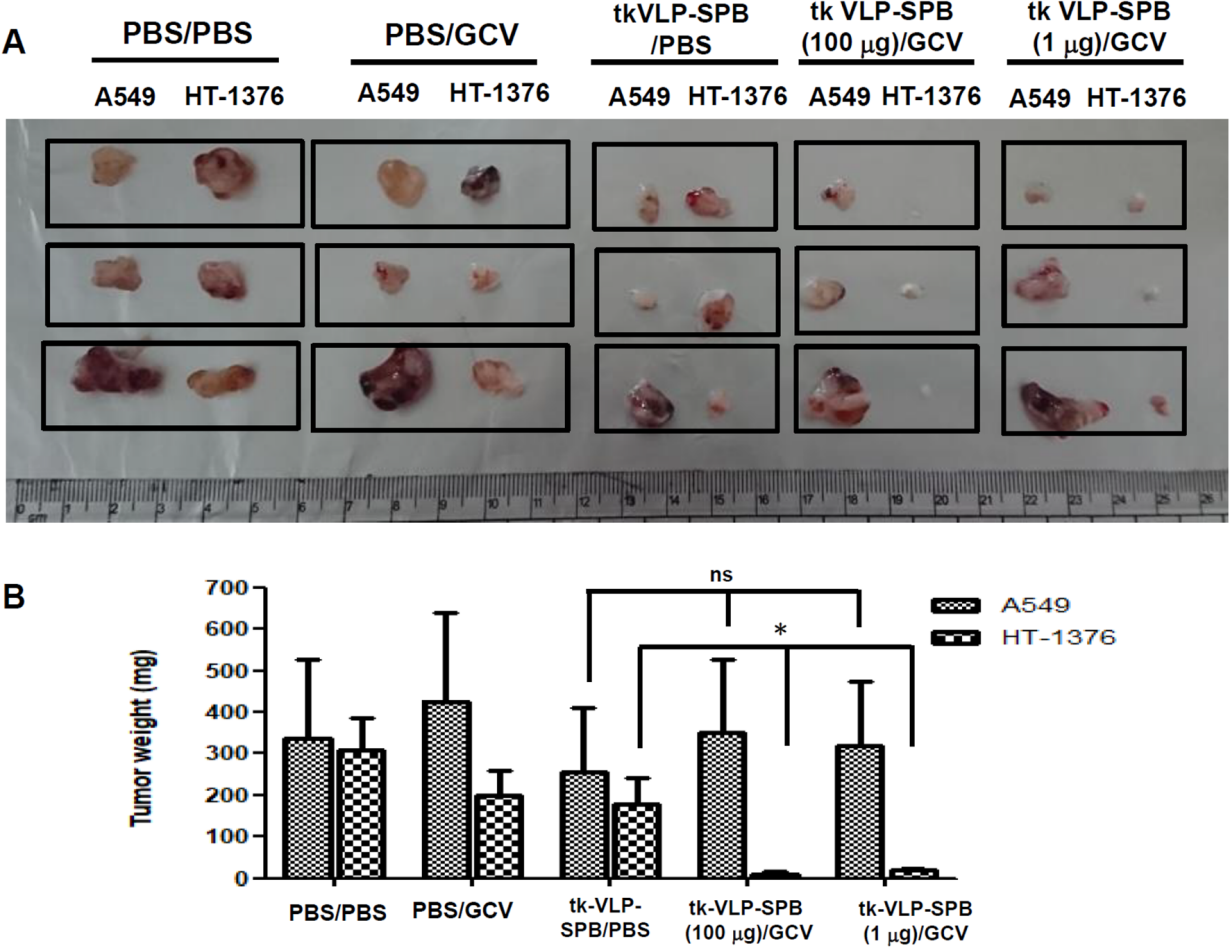
Specific inhibition of bladder cancer growth by tk-JCPyV VLP-SPBs in a xenograft mouse model. Mice were injected with A549 and HT-1376 cells in the left and right dorsal regions, respectively. The mice were divided into five groups and injected with PBS (PBS/PBS), GCV (PBS/GCV), tk-JCPyV VLP-SPB/PBS, tk-JCPyV VLP-SPB (100 μg)/GCV, or tk-JCPyV VLP-SPB (1 μg)/GCV. JCPyV VLP-SPB was injected into the tail vein, and GCV was administered by intraperitoneal injection. Injections were given once every three days for a total of 12 injections. Mice were anesthetized and euthanized with isoflurane, and the tumors were collected and weighed. (**A**) Photographs of tumors in the five groups. (**B**) Tumor weights in the five groups. *: *P* < 0.05 was considered statistically significant and is indicated by an asterisk (*). ns: not significant.

We subcutaneously injected A549 lung cancer cells and HT-1376 bladder cancer cells into the left and right sides, respectively, of the same mouse. One week later, the mice were randomly divided into five groups, 3 mice in each group: the control group and groups injected with PBS (PBS/PBS), GCV (PBS/GCV), tk-JCPyV VLP-SPBs/PBS, tk-JCPyV VLP-SPBs (100 μg)/GCV, and tk-JCPyV VLP-SPBs (1 μg)/GCV. Different concentrations of 1 μg and 100 μg of tk-JCPyV VLP-SPB were injected to the tail vein, and GCV was administration by intraperitoneal injection (tk-JCPyV VLP-SPB/GCV). Mice administrated with PBS/PBS and PBS/GCV were used as GCV toxicity control; mice treated with tk-JCPyV VLP-SPB without GCV administration (tk-JCPyV VLP-SPB /PBS) were used as control for tk-JCPyV VLP-SPB/GCV. As shown in Fig. 5, injection of 100 µg and 1 µg of tk-JCPyV VLP-SPBs combined with GCV significantly inhibited the growth of tumors from only HT-1376 bladder cancer cells but not those of A549 lung cancer cells (Fig. 5A, tk-JCPyV VLP-SPB/GCV, the fourth and fifth panels, respectively). The growth of A549 lung cancer cells injected with tk-JCPyV VLP-SPBs combined with GCV group (Fig. 5A, left side of fourth and fifth panels) did not differ from that of tumors in the tk-JCPyV VLP-SPB/PBS control (Fig. 5A, left side of the third panel), demonstrating the high specificity of tk-JCPyV VLP-SPBs for bladder cancer. In addition, treatment with a reduced tk-JCPyV VLP-SPB concentration of 1 µg in combination with GCV significantly inhibited the growth of tumors from HT-1376 bladder cancer cells with approximately the same effect as 100 µg of tk-JCPyV VLP-SPB (Fig. 5A, right side of the fourth and fifth panels). There were no significant differences in the size of subcutaneous A549 and HT-1376 tumors in the control groups (PBS/PBS, PBS/GCV, tk-JCPyV VLP/PBS, Fig. 5A,B). The body weight of the mice showed no significant difference during the experiment (data not shown). These results further demonstrated that the specific targeting of JCPyV VLPs can be altered through conjugation to a specific peptide in an in vivo animal model through the tail vein administration.

## Discussion

In the present study, we conjugated a bladder cancer-specific peptide (i.e., SPB) with JCPyV VLPs and confirmed that the resulting JCPyV VLP-SPBs bound specifically to bladder cancer cells. JCPyV VLP-SPBs effectively delivered the tk suicide gene, which is cytotoxic to bladder cancer cells, only to bladder cancer cell lines and not to cell lines originally demonstrated to be susceptible to JCPyV VLPs, such as lung cancer and neuroblastoma cell lines. The xenograft mouse model also showed that JCPyV VLP-SPB only inhibited the growth of bladder tumors, while the growth of lung cancer cells on the contralateral side was unaffected. Therefore, the present study demonstrated that conjugation with a specific peptide can redirect JCPyV VLPs to specific tissues and can also significantly increase the transduction specificity and efficiency of JCPyV VLPs as a gene therapy vector.

VLPs have no viral genes but retain the characteristics of virus-specific binding to host cells, thus functioning as an important type of gene delivery vector in the development of gene therapy^32^. However, the ease of preparation and the cost of gene delivery vectors are also important factors to consider in vector development. JCPyV VLPs are produced in *E. coli* and self-assemble into VLPs, which can be easily purified by sucrose gradient centrifugation; thus, they are easy to obtain in a cost-effective manner^10^. During assembly, JCPyV VLPs can package DNA molecules of up to approximately 9.4 kb in size, which is sufficient to address current limitations regarding the size of genes required for treatments^22^. JCPyV VLPs are an efficient gene therapy vector also because they can deliver and protect the entry of genes into both dividing and nondividing cells^23^. To achieve tissue specificity, we previously used JCPyV VLPs to deliver tissue-specific promoters to specifically express a gene of interest in a particular cell; these promoters included the lung cancer-specific *SPB* promoter^17,18^, prostate cancer-specific *PSA* promoter^19^, and *MUC1* promoter, which is overexpressed in bladder cancer^16^. In the current study, we further conjugated JCPyV VLPs with a bladder cancer cell-specific binding peptide for investigation of specific targeting. This conjugation allowed JCPyV VLPs to target bladder cancer cells and subsequently inhibited their growth, demonstrating that conjugation with a specific peptide increased the therapeutic specificity of JCPyV VLPs. In addition, the results also show a 100-fold increase in efficiency in terms of tumor weight in terms of therapeutic efficiency. Therefore, JCPyV VLPs can be guided by a cell surface marker binding peptide for the specific delivery and transduction of a gene of interest expressed by a tissue-specific promoter in a targeted cell type. VLPs retain the characteristics of virus-specific binding to host cells, but are allowed for genetic or chemical modification to alter the tropism or increase their binding efficiency to their nature host cells^33,34^. For example, the specific cellular targeting of MS2 VLPs are demonstrated through attachment of a tissue specific nucleic acid aptamers^35^. Conjugation of folic acid on VLP derived from truncated hepatitis B virus core antigen (tHBcAg) increases the specificity and efficacy of the drug delivery^36^. By genetic incorporation of cancer specific peptides to the hybrid adeno-associated virus/phage (AAVP) particles results in an increase gene transfer efficiency^37^. In the current study, we demonstrated that the tropism of JCPyV VLP was altered after conjugated with the specific peptide for bladder cancer, SPB.

Among the types of bladder cancers, urothelial carcinoma is characterized by high recurrence, and approximately 10–20% of cases progress to MIBC and metastasis. Many drugs have been developed for the treatment of metastatic bladder cancer, but the clinical response remains limited^29^. The HSV-tk/GCV system is one of the most commonly used and studied prodrug systems. Viral thymidine kinases convert guanosine analog GCV into GCV monophosphate which can be easily converted into toxic triphosphate GCV by cellular kinases. When triphosphate GCV is incorporated into replicating DNA, a termination of DNA replication and cell apoptosis will occur. The human cellular kinases exhibit much lower affinity to convert GCV compared to HSV-tk. Only cells receiving HSV-tk gene can convert GCV (prodrug) to monophosphate GCV, and ultimately toxic triphosphate GCV (drug)^38,39^. In addition, a bystander effect which can amplify the toxicity of triphosphate GCV is also observed in HSV-tk/GCV system, which makes HSV-tk/GCV system an attractive strategy for cancer therapy^39,40^. However, the clinical trial of HSV-tk/GCV system did not show promising effect for patients with glioblastoma multiforme and advanced hepatocellular carcinoma^41,42^. The gene transfer efficiency is one of the challenges in these systems. In the present study, HSV-tk gene was packaged in JCPyV VLPs and conjugated with specific bladder cancer peptide, SPB. The tk-JCPyV VLP-SPBs were administered to mice via the tail vein and effectively inhibited the growth of bladder tumor nodules in the mice. These results demonstrated that after tk-JCPyV VLP-SPB entered the circulation; it specifically targeted bladder cancer cells. The findings also suggested that JCPyV VLPs can be applied through i.v. injections for the treatment of metastatic tumors if conjugated with a tissue-specific ligand. However, delivery of a large quantity of JCPyV VLP-SPB may also have off-target effects, as shown in cytotoxicity tests in Fig. 3A,B. Furthermore, most people are seropositive to JCPyV, the use of JCPyV VLPs as a delivery vector may result in a decreased efficiency. In order to avoid the immune elimination, a modification of the surface of JCPyV VLPs may be a strategy for developing the JCPyV VLPs as a human gene delivery vector.

In summary, JCPyV VLPs can function as a gene therapy vector and can package a gene of interest driven by a tissue-specific promoter. The present results further demonstrated that conjugation with specific peptides allows the original tropism of JCPyV VLPs to be altered, thereby redirecting the encapsidated genes for expression in specific tissues and enhancing the gene transduction efficiency. Therefore, JCPyV VLPs could be designed as a flexible gene delivery vector targeted to different cell types for a variety of therapeutic purposes.

## Methods

### Cell lines

All cell lines were purchased from the Bioresource Collection and Research Center, Taiwan. The culture medium for the human bladder cancer cell lines HT-1197 and HT-1376 was minimum essential medium supplemented with l-glutamine, nonessential amino acids, sodium pyruvate, fetal bovine serum, and penicillin/streptomycin antibiotic solution (Thermo Fisher Scientific, Cambridge, MA, USA). Human lung adenocarcinoma (A549) and neuroblastoma (IMR-32) cell lines were cultured in culture dishes (Guangzhou Jet Bio-filtration Co. Ltd. Guangzhou, China) with Dulbecco’s modified Eagle medium supplemented with nonessential amino acids, sodium pyruvate, fetal bovine serum, and penicillin/streptomycin antibiotic solution (Thermo).

### SPB binding to bladder cancer cells

The SPB peptide (CSNRDARRC), which has specificity for bladder cancer^28^, was synthesized by AngeneBiotech (Taipei, Taiwan). The red fluorescent tetramethylrhodamine (TAMRA) molecules were simultaneously added to the N-terminus of SPB in the process of synthesis (AngeneBiotech), which is named as TAMRA-SPB. To test the binding of TAMRA-SPB to HT-1376 cells, 5 × 10^5^ cells were seeding onto a coverslip (Marienfeld, Lauda-Königshofen, Germany) placed at 4 °C and allowed to stand for 10 min. The culture medium was removed, and the cells were washed twice with cold PBS. After the addition of 20 µM, 50 µM, or 100 µM TAMRA-SPB, the mixture was placed at 4 °C for 30 min, and the cells were washed three times with 4 °C PBS. The cells were then mounted using a mounting solution containing 0.1% DAPI (Sigma-Aldrich, D9542) and observed under a confocal microscope (Olympus, FluoView 1200) with 400X magnification.

### Testing the specificity of the SPB for bladder cancer cells

5 × 10^5^ cells of HT-1376 cells were placed at 4 °C and allowed to stand for 10 min. The culture medium was removed, and the cells were washed twice with cold PBS. After the addition of 0.002, 0.02, 0.2, 2, 20, or 200 μM SPB to TAMRA-SPB (20 μM), HT-1376 cells were added to the mixture, placed at 4 °C for 30 min, and washed three times with 4 °C PBS. Cells were mounted using a mounting solution containing 0.1% DAPI and observed using a confocal microscope (Olympus, FluoView 1200) with 400X magnification.

### Preparation of the JCPyV VLPs

Preparation of the JCPyV VLPs was performed according to our previous reports^13–18^. Briefly, the VP1 sequence of JCPyV (GenBank:#U61771) has been cloned into △pFlag plasmid (△pFlag -JCPyV VP1). pUMVC1-tk (Aldevron, Fargo, ND, USA) under CMV promoter for tk expression, and △pFlag -JCPyV VP1 for VP1 expression, were transformed into the *E. coli* JM109. The transformed *E. coli* strains were grown in Luria Broth (LB) medium containing kanamycin and ampicillin to select for pUMVC1-tk and △pFlag-JCPyV VP1, respectively. JCPyV VP1 protein expression was induced by the addition of 0.5 mM (final concentration) isopropyl b-D-1-thiogalactopyranoside (IPTG) for 8 h at 30 °C. Expression of JCPyV VP1 was able to self-assembled into a virus-like particle (VLP), and simultaneously package the second plasmid, pUMVC1-tk, present in the *E. coli*. The *E. coli* lysate, which contains pUMVC1-tk plasmids packaged in JCPyV VLPs, was collected and subjected to 20% sucrose cushion, followed by 10% to 30% sucrose gradient centrifugation. Fractions with hemagglutination activity, which indicates an integrity form of VLPs^9,11^, were collected and dialyzed against Tris-buffered saline (10 mm Tris–HCl, pH 7.4, 150 mm NaCl). The VLPs were concentrated by using a Centricon filter (Millipore, Billerica, MA, USA). The purified VLPs containing pUMVCl-tk were then designates as tk-JCPyV VLPs. The purity and stability of tk-JCPyV VLPs were determined by SDS-PAGE and hemagglutination activity analysis, respectively.

### Conjugation of SPB peptide to JCPyV VLPs

One mL of JCPyV VLPs (1 mg/mL) mixed with 40 µL of Sulfo-SMCC (sulfosuccinimidyl 4-(N-maleimidomethyl)cyclohexane-1-carboxylate, Thermo Fisher Scientific) (4.8 mg/mL) for 30 min at room temperature. The free Sulfo-SMCC was removed by a desalting column (Zeba Spin Desalting Columns, 7K MWCO, Thermo Fisher Scientific). The desalted JCPyV VLPs was mixed 100 µL of SPB peptide (1 mM) for 30 min. The free SPB peptide was removed by the desalting column. HA was performed for evaluation the integrity of the JCPyV VLP-SPB.

### Testing the cell binding specificity of JCPyV VLP-SPBs

5 × 10^5^ cells of A549, IMR-32, HT-1197, and HT-1376 cells were separately treated with PBS or with 1, 3, or 10 μg/mL JCPyV VLP-SPB-TAMRA; incubated at 37 °C for 20 min; and washed three times with PBS. Slides were mounted using a mounting solution containing 0.1% DAPI and observed using a fluorescent microscope (Carl Zeiss, Thornwood, NY) with 200X magnification.

### Cytotoxicity assay

1 × 10^4^ Cells were cultured in 96-well plates and treated with tk-JCPyV VLPs or tk-JCPyV VLP-SPBs at different concentrations. Next, 20 μg/mL GCV (InvivoGen) was added, and the cells were cultured at 37 °C for 72 h. The cytotoxicity of the tk suicide gene to the cancer cells was analyzed using a Cell Counting Kit-8 (CCK-8; Sigma-Aldrich, St. Louis, MO, USA) according to the manufacturer’s instructions.

### Analysis of specific inhibition of human bladder tumor growth by tk-JCPyV VLP-SPBs in a xenograft mouse model

The Institutional Animal Care and Use Committee of Chung-Shan Medical University approved the animal experiments (IACUC permit Number: 2046). All animal procedures were performed according to approved protocols and in compliance with the recommendations for proper care and use of laboratory animals^43^. Four-week-old male nude mice were purchased from BioLASCO Taiwan Co., Ltd. The mice were injected with 10^6^ A549 and HT-1376 cells in the left and right dorsal region, respectively. One week later, the mice were randomly divided into five groups, 3 mice in each group: the control group and groups injected with PBS (PBS/PBS), GCV (PBS/GCV), tk-JCPyV VLP-SPBs/PBS, tk-JCPyV VLP-SPBs (100 μg)/GCV, and tk-JCPyV VLP-SPBs (1 μg)/GCV. JCPyV VLP-SPBs were injected into the tail vein, and GCV was administered by intraperitoneal injection. Injections were given once every three days for a total of 12 injections of PBS (100 µL), tk-JCPyV VLP-SPBs, or GCV (300 mg/kg). One month later, the mice were anesthetized and euthanized with isoflurane, and the tumors were collected and weighed.

### Statistical analysis

Statistical significance was determined using GraphPad Prism 5.0 software (GraphPad Software, San Diego, CA, USA). Data were analyzed using the Kruskal–Wallis test. *P* values of less than 0.05 were considered statistically significant and indicated with an asterisk (*).

## Abbreviations

GCV: Ganciclovir
SPB: Specific peptide for bladder cancer
TAMRA: Tetramethylrhodamine
tk: Thymidine kinase
VLP: Virus-like particle
